# Sensitivity Characteristic Analysis of Adsorbent-Mixed Carbon Nanotube Sensors for the Detection of SF_6_ Decomposition Products under PD Conditions

**DOI:** 10.3390/s131115209

**Published:** 2013-11-07

**Authors:** Xiaoxing Zhang, Chenchen Luo, Ju Tang

**Affiliations:** State Key Laboratory of Transmission & Distribution Equipment and Power System Safety and New Technology, Chongqing University, Chongqing 400040, China; E-Mails: luocc89@126.com (C.L.); cqtangju@vip.sina.com (J.T.)

**Keywords:** SO_2_, H_2_S, PD, carbon nanotube, adsorbent, gas sensor, gas response

## Abstract

Sulfur hexafluoride (SF_6_) gas decomposition results from the energy produced by partial discharge (PD). The detection of SO_2_ and H_2_S content, as important characteristic components of the decomposition products, is significant in the determination of the insulation level of SF_6_ gas and the inside insulation faults of gas-insulated equipment. A number of gas sensors use carbon nanotubes (CNTs). However, the applications of these sensors are limited by their low intrinsic sensitivity. In this paper, an adsorbent-mixed carbon nanotube gas sensor is proposed to improve the detection of SO_2_ and H_2_S concentrations. The sensitivity of adsorbent-mixed carbon nanotube gas sensors to SO_2_ and H_2_S at 100 ppm was investigated experimentally. The effect of the mixing ratio on the gas sensitivity characteristic and mechanism of response was also studied. The results show that compared with intrinsic CNTs gas sensors, the gas sensor featuring adsorbent-mixed CNTs has significantly higher sensitivity and responsiveness to SO_2_ and H_2_S. The resistance-change rate of SO_2_ and H_2_S increased to 33.7% and 50.9% from 0.96% and 12.9%, respectively. Moreover, the resistance-change rate and gas concentration satisfy a linear relationship. The sensor has good repeatability and stability.

## 1. Introduction

With the development of the power industry, gas-insulated switchgear (GIS) has become more widely used in the power grid for its advantages of high reliability, small volume, and simple maintenance. Sulfur hexafluoride (SF_6_) is used in GIS for its good insulating performance and arc extinction properties [1–4]. The manufacture, transportation and installation process inevitably result in defects in GIS equipment. These defects include metal burrs, protrusions, or suspended particles. In long-term operation, these latent defects could cause partial discharge (PD). SF_6_ may undergo a decomposition reaction when it reacts with water and oxygen to produce a series of compounds, such as HF, SO_2_, H_2_S, SO_2_F_2_, and SOF_2_[5,6]. These products will accelerate insulation aging and corrode metal surfaces, finally resulting in GIS faults [4]. As important SF_6_ decomposition characteristic component gases, the detection of variations in the concentrations of SO_2_ and H_2_S is significant to determine the insulation level of SF_6_ gas and the development conditions of PD. Moreover, such detection lays a solid foundation for further diagnosis and assessment of GIS equipment operating status.

Carbon nanotubes (CNTs) have a rich pore structure, large specific surface area, and good adsorption and desorption properties. Research on CNT usage for gas detection has focused on the improvement of the sensitivity and stability of CNT gas sensors to different gases. Studies have shown that electrons are exchanged between the CNT and the adsorbed gas, which aids in characterizing different electrical properties [7–10]. Based on these properties, researchers have applied various modification methods to change the electronic structure of CNTs to improve their sensitivity and selectivity to different gases [11–14].

In this work, an adsorbent-mixed CNT gas sensor based on the intrinsic CNT gas sensor is proposed to detect some characteristic SF_6_ decomposition products in GIS, study the gas responses of the CNT sensor to SO_2_ and H_2_S, and discuss the mechanism of these gas responses. The results show that the adsorbent mixed into multi-walled CNTs (MWNTs) increases the sensitivity of the sensor to SO_2_ and H_2_S.

## 2. Experimental Section

### 2.1. Structure and Characteristics of 4 Å Zeolite

**Figure 1.**
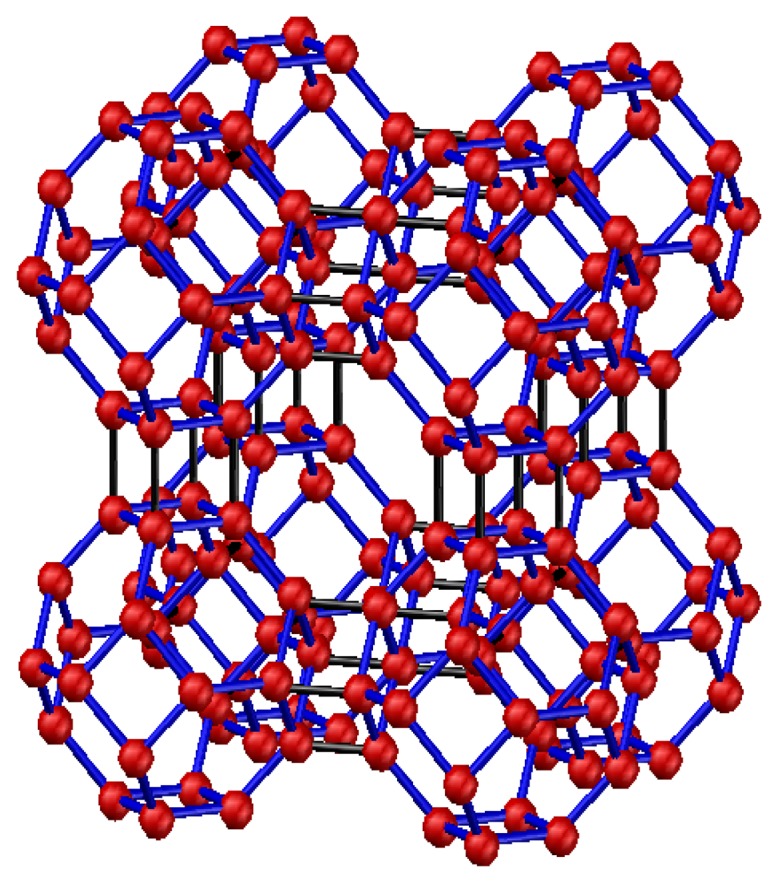
Structure model of 4 Å molecular sieve.

The 4 Å molecular sieve was selected in this paper. Synthetic zeolites are a kind of aluminosilicate with a microporous cubic lattice. As a type of Å zeolite, the 4 Å molecular sieve is named after the effective aperture of its molecular structure, which is 4 Å. The structure of 4 Å zeolite, which is similar to the structure of NaCl crystals, is shown in Figure 1.

The basic structure of 4 Å zeolite includes a silicon-oxygen and an aluminum-oxygen tetrahedron. Four oxygen atoms surround the central silicon (or aluminum) atom in each tetrahedron. Two adjacent silicon-oxygen (or aluminum-oxygen) tetrahedrons constitute the chamfered octahedral (β cage) by the sharing an oxygen atom. Each vertex of the cube has a β cage, and eight β cages connect to form a large α cage in the center. In sum, each α cage is surrounded by eight β cages and six α cages. The α and β cages are interconnected by six rings, whereas α and α cages are interconnected by eight rings. Thus, a plurality of wide cavities and channels is formed in this kind of structure. The ideal cell of Å zeolite is Na_96_(Al_96_Si_96_O_384_)·216 H_2_O, which is equivalent to eight β cages.

In the tetrahedral framework structure of zeolite, aluminum is trivalent, and four oxygen atoms surround an aluminum atom. That is, the aluminum-oxygen tetrahedron has a negative charge. To maintain the charge balance, metal cations offset the negative charge. The metal cations in 4 Å zeolite are sodium ions. The sodium ions and oxygen atoms form a stable structure.

The particular zeolite structure is thus characterized by a large surface area and strong surface field. In the tetrahedron, the Na^+^ and anion skeleton form dipoles, which compensate for the negative charge. 4 Å zeolite is a kind of polar substance that more easily absorbs polar substances.

Relevant literature and tests have not determined to date the conductive properties of the adsorbent. Thus, the conductivity of the sensors in this experiment is unrelated to the conductivity of the adsorbent.

### 2.2. Preparation of MWNT Sensors

In this paper, CNTs were prepared through chemical vapor deposition. The CNTs have a multi-wall structure with purity of more than 95%. The tube diameter ranges from 20 to 30 nm, whereas the tube lengths range from 10 to 30 μm. The specific surface area of 4 Å molecular sieve is about 800 g/m^2^ in the paper. Filtered by 5,000 mesh sub-sieve, the ground 4 Å molecular sieve has micrometer dimensions, with a powder particle diameter of approximately 1 μm.

First, the MWNTs and the ground micrometer size 4Å molecular sieve were weighed at different mass ratios. In this paper, the following mass ratios were selected: 1:1, 3:1, 5:1, 10:1, and 20:1. The MWNTs were placed in anhydrous ethanol and the mixed solution was dispersed for 90 min using an ultrasonic oscillator to obtain a uniformly dispersed suspension. A certain amount of the solution was taken and placed it in anhydrous ethanol. The mixed solution was then dispersed several times for 60 min to obtain a clearer solution.

The MWNT sensor substrate is made of printed circuit boards. Copper interdigital electrodes are etched in the substrate. Foil thickness is approximately 30 μm, whereas the width and spacing of the electrodes are both 1 mm.

Trace solution was dropped onto the surface of the interdigital electrodes, until the initial resistance values of the sensors meet the needs, and then placed in an oven at 80 °C to prepare a uniform, dense, and smooth film to serve as the gas membrane for the detection of characteristic SF_6_ decomposition products.

**Figure 2.**
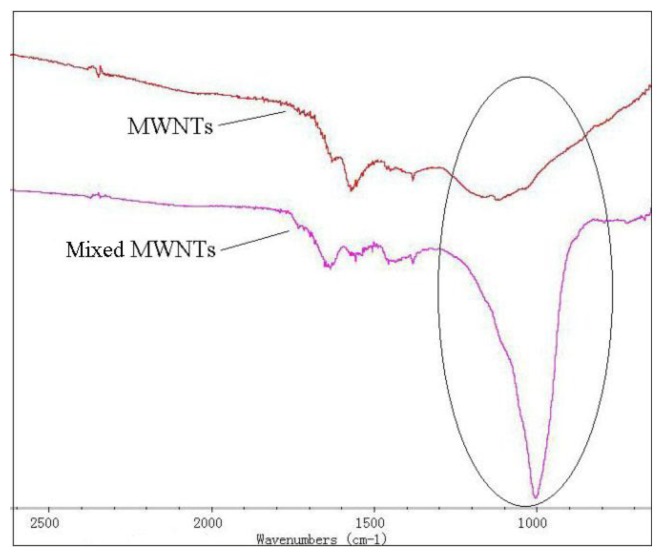
Infrared absorption spectra of MWNTs and mixed MWNTs.

**Figure 3.**
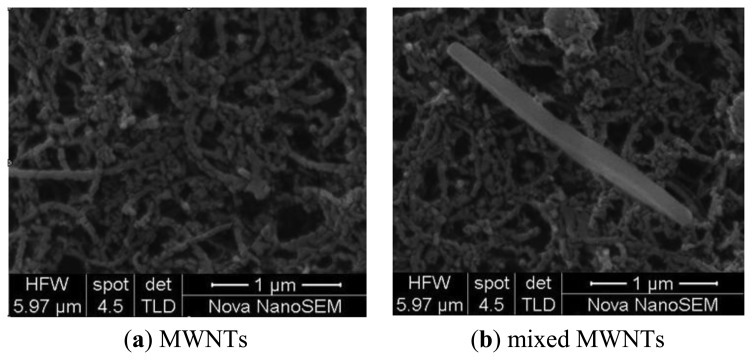
SEM images of MWNTs.

Figure 2 shows a comparison of the intrinsic MWNTs and the adsorbent-mixed MWNTs recorded using a Nicolet 5DXCFT-IR infrared spectrometer. From the infrared spectra in Figure 2, an evident silicon-oxygen bond absorption peak is observed at 1,000 to 1,100 cm^−1^ (circled in Figure 2) of in the adsorbent-mixed MWNTs. Silicon-oxygen bonds exist in the adsorbent, but not in MWNTs. This proves the adsorbent is mixed into the MWNTs. Figure 3a,b shows the SEM images of the intrinsic MWNTs and adsorbent mixed MWNTs, respectively.

According to SEM image, the 4 Å zeolite powders are present in the mixture. Meanwhile, it could be observed that the powder has micrometer dimensions.

## 3. Results and Discussion

### 3.1. Procedures for Detecting SF_6_ Decomposition Products

**Figure 4.**
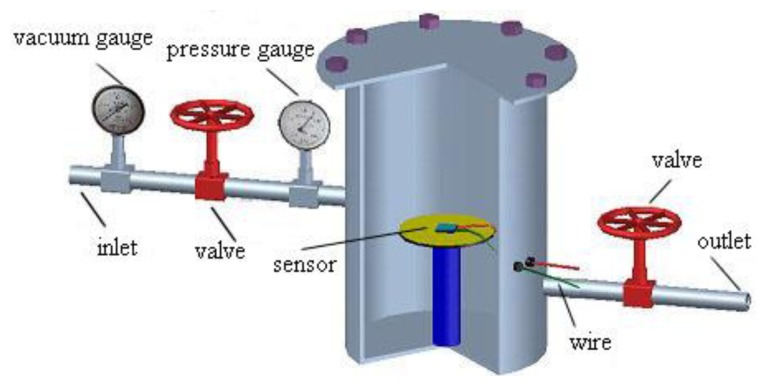
Structure of the gas chamber used for testing sensors.

The prepared MWNT sensor was placed in a sealed chamber, as shown in Figure 4. The MWNT sensor was then connected to an impedance analyzer through a wire. Finally, screws were used to seal the gas chamber.

The experimental steps were as follows:

(1) The inlet valve was closed, the vacuum gauge and the outlet valves were opened, and the vacuum pump was switched on to pump air from the cylinder. The ball valves and vacuum pump were then closed after the cylinder became a vacuum. The number displayed on the vacuum gauge was observed after 12 h to verify whether the gas chamber pressure was stable. Subsequently, the initial resistance value *R*_0_ of the MWNT sensor shown in the impedance analyzer at this time was recorded.
(2) Gaseous SF_6_ decomposition product was passed into the sealed chamber through the inlet valve. Meanwhile, the resistance values *R* of the MWNT sensor were recorded until no change was observed. The resistance-change rate of the sensor, which is called the response of the sensor, was calculated as:
  (1) $$\rho =\left(R-R_{0}\right)/R_{0}\times 100\%$$
  where *R* is the resistance value of the sensor after the injection of SF_6_ decomposition product gas, and *R*_0_ is the initial resistance value of the sensor under vacuum.
(3) When the test was finished, SF_6_ decomposition product gas was pumped from the cylinder and N_2_ gas was passed into the cylinder to ensure that there was no residual gas present. The above steps were repeated to test the other sensors.

### 3.2. MWNT Sensor Response to SO_2_ and H_2_S at 100 ppm

**Figure 5.**
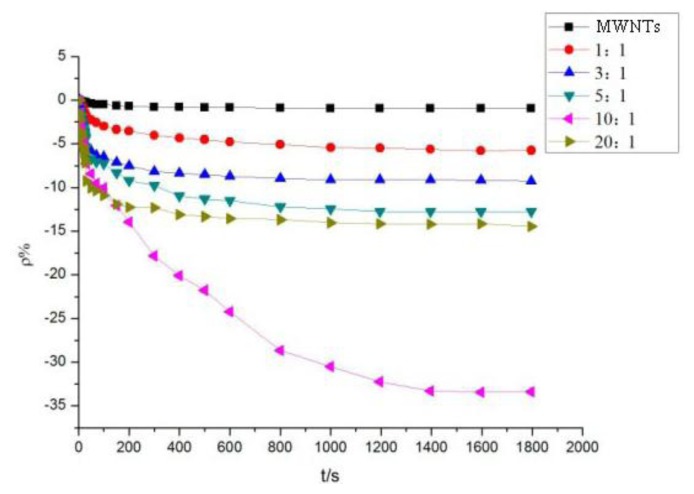
MWNT sensor response to SO_2_.

**Figure 6.**
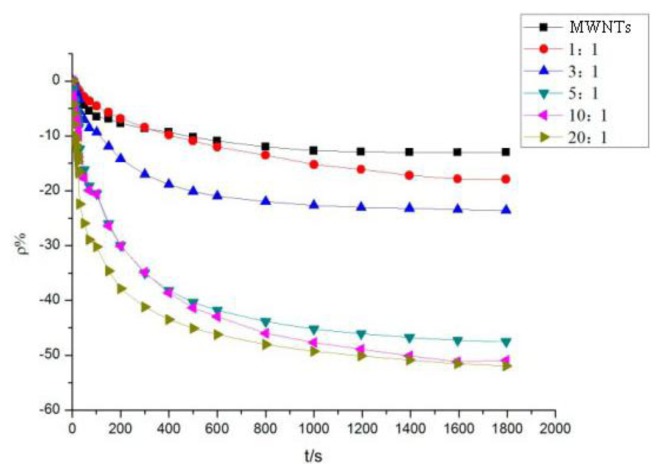
MWNT sensor response to H_2_S.

The method mentioned in Section 3.1 was used to test the intrinsic and mixed MWNT sensors. Various sensors were used to detect SO_2_ and H_2_S at a concentration of 100 ppm. Figure 5 shows the response curves of the MWNT sensors to SO_2_ gas, whereas Figure 6 shows the response curves of the MWNT sensors to H_2_S gas.

Figure 5 shows that compared with the intrinsic MWNT gas sensor, the gas sensor featuring adsorbent-mixed MWNTs had a greater resistance-change rate. Consequently, the response curve to SO_2_ became steeper. When SO_2_ is detected at a concentration of 100 ppm, the resistance-change value of the intrinsic MWNT sensor was found to be 0.96%. Mixed with different proportions of adsorbent, the resistance-change value of gas sensors increased at varying degrees. Therefore, the adsorbent mixed to MWNTs improved the sensitivity of MWNTs to SO_2_ gas. With a mass ratio of 10:1, the resistance-change rate of the adsorbent-mixed MWNT sensor to SO_2_ increased to 33.7%, which is 35 times that of the intrinsic MWNT sensor. The adsorption capacity of the adsorbent-mixed MWNT sensor at a ratio of 10:1 increased significantly, and the response rate was improved markedly.

A similar observation can be made from Figure 6. Compared with the intrinsic MWNT gas sensor, the gas sensor featuring the adsorbent-mixed MWNTs possessed a higher resistance-change rate to H_2_S. When detecting H_2_S at a concentration of 100 ppm, the resistance-change value of the intrinsic MWNT sensor was found to be 12.92%. Mixed with different proportions of adsorbent, the resistance-change value of gas sensors increased at varying degrees. With the mass ratios of 5:1, 10:1, and 20:1, the resistance-change values of the adsorbent-mixed MWNT sensors to H_2_S increased to 47.6%, 50.9%, and 51.8%, respectively. These values are approximately four times the resistance-change rate of the intrinsic sensor. The sensitivity and response rate of the adsorbent-mixed MWNT sensor evidently increased.

**Figure 7.**
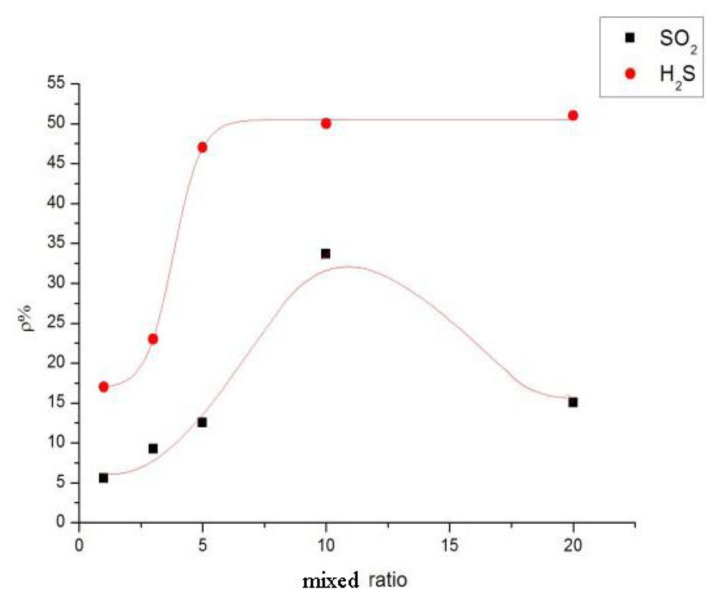
MWNT sensor response to SO_2_ and H_2_S.

The different sensor response results of mixed ratios are shown in Figure 7. At a mass ratio of 10:1, the resistance-change rate of the adsorbent-mixed MWNT sensor to SO_2_ is significantly greater than that of other ratios. At mass ratios of 5:1, 10:1, and 20:1, the resistance-change values of the adsorbent-mixed MWNT sensors to H_2_S were all large. Thus, sensors with different mixed ratios were selected to determine the selectivity of the two gases according to this feature.

### 3.3. Selectivity of Different Sensor Ratios to SO_2_ and H_2_S at 100 ppm

**Figure 8.**
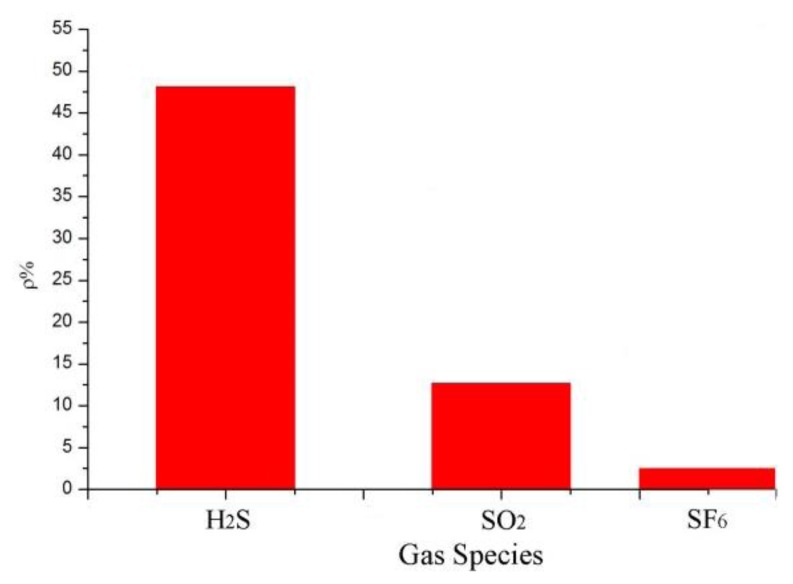
Sensor selectivity of MWNT sensor response to SO_2_ and H_2_S.

According to the analytical results in Section 3.2, the sensor with a mass ratio of 5:1 was selected to detect SO_2_ and H_2_S at 100 ppm and the results were then analyzed. SF_6_ is the main gas in GIS, and SO_2_ and H_2_S are produced by the decomposition of SF_6_ under certain conditions. Thus, the influence of SF_6_ and the sensor response to SF_6_ as a background gas have to be considered. In accordance with the test method in Section 3.1, the gas responses of the MWNT sensors were measured at 100 ppm SO_2_, 100 ppm H_2_S, and 99.999% SF_6_. The results are shown in Figure 8. The resistance-change values of the adsorption-mixed sensor at 100 ppm SO_2_, 100 ppm H_2_S, and 99.999% SF_6_ were 48%, 12%, and 2.7%, respectively. The results illustrate that the mixed sensor with a ratio of 5:1 had good selectivity to H_2_S gas and is therefore suitable for detecting H_2_S gas, which is a SF_6_ decomposition product in GIS.

### 3.4. MWNT Sensor Response to SO_2_ and H_2_S at Different Concentrations

**Figure 9.**
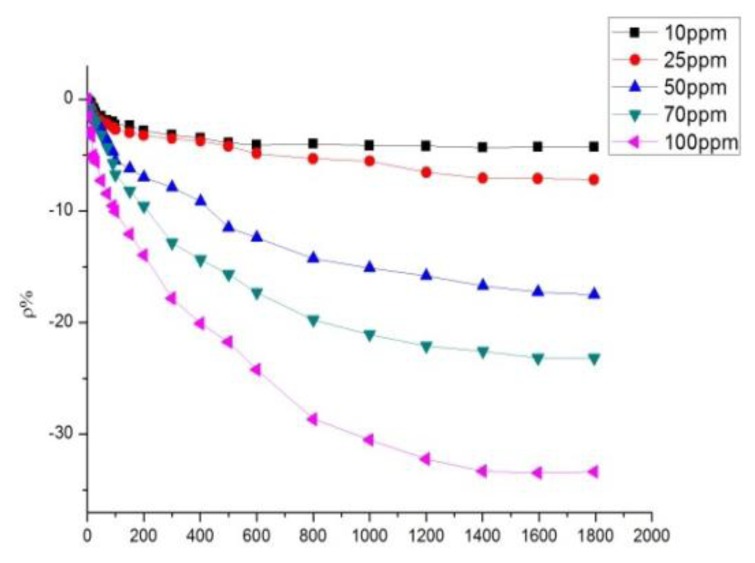
MWNT sensor response to different concentrations of SO_2_.

According to the analytical results in Section 3.2, the sensor with a mass ratio of 10:1 was selected to detect SO_2_ at different concentrations, whereas that with a mass ratio of 5:1 was selected to detect H_2_S at different concentrations. The sensor with a mass ratio of 10:1 was used to detect SO_2_ at different concentrations of 10, 25, 50, 70, and 100 ppm. The response curves are shown in Figure 9.

**Figure 10.**
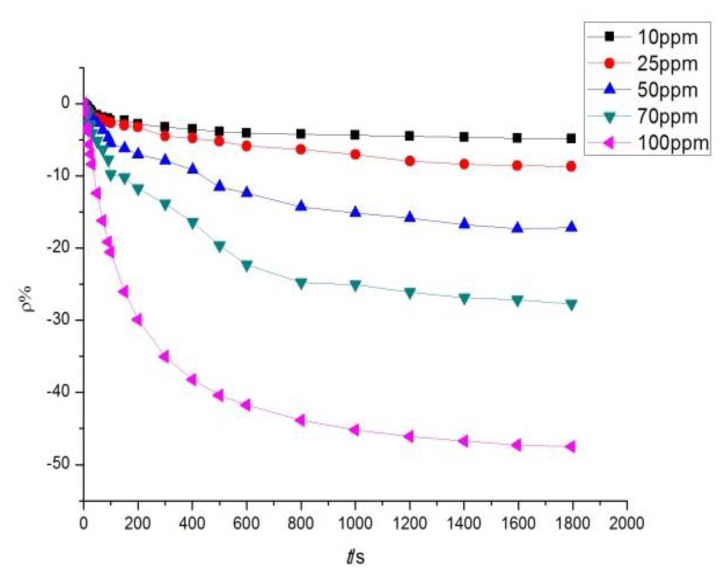
MWNT sensor response to different concentrations of H_2_S.

**Figure 11.**
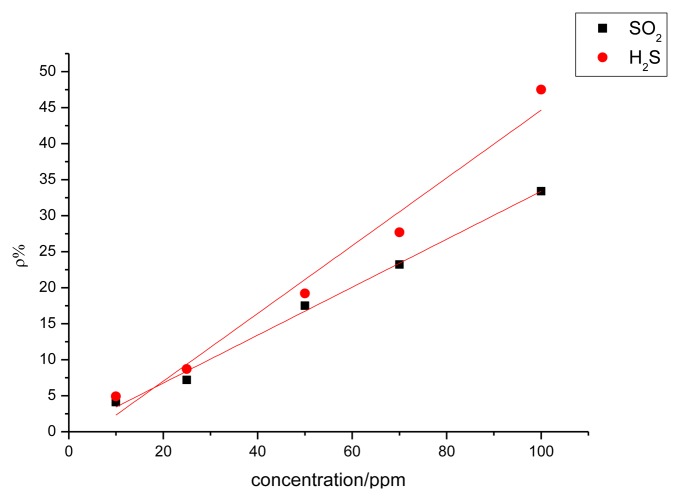
Linear relationship of MWNT sensor gas sensing response with different concentrations of SO_2_ and H_2_S.

The sensor with a mass ratio of 5:1 was used to detect H_2_S at different concentrations of 10, 25, 50, 70, and 100 ppm. The response curves are shown in Figure 10. Figures 9 and 10 illustrate that a greater concentration of SO_2_ and H_2_S gas results in higher sensor response. Figure 11 describes the relationship between gas concentration and resistance-change rate. The relation between the concentration of the two gases and resistance-change rate satisfies the specified linear relation under a specific concentration range (10 to 100 ppm in this paper). The linear correlation coefficients *R*^2^ are 0.994 and 0.969, respectively.

As shown in Figure 11, the sensor resistance-change rate can be used to determine the size of the measured gas concentration. The capability of the sensor to detect low concentrations of SO_2_ and H_2_S gas is of certain practical value. In Figure 11, the slopes of the two curves represent the sensitivity of the sensors. Figure 11 illustrates that the sensitivity of the sensor to H_2_S is greater than to SO_2_. As is known to all, H_2_S is a strong reducing gas, while the reduction capability of SO_2_ is weaker than that of H_2_S. The charge transfer capacity of carbon nanotubes to H_2_S should be stronger than to SO_2_, that is to say, the response to H_2_S is greater than to SO_2_.

### 3.5. MWNT Sensor Test of Desorption and Repeatability

The test was repeated according to the resistance value of the tested sensors in Section 3.2 to restore them to the initial value via a desorption treatment. Pure N_2_ was injected into the tested sensor and the sensor was irradiated using UV light to complete the desorption test. The desorbed sensor, which was again ready for gas detection, was then tested.

After N_2_ treatment to flush the tested sensor, sensor resistance can generally remain near the initial value. However, after several tests, a small amount of residual gas accumulates on the surface of the sensor, which causes a “poisoning” phenomenon. As a result, a certain degree of reduction in sensor sensitivity is observed. UV light is required for the desorption treatment of residual gas, which consequently restores the high sensitivity of the sensor. By irradiating with UV light, the residual gas absorbs energy, which enables it to “escape” from the “trapped” state to the point where almost no residual gas remains on the surface of the tested sensor.

**Figure 12.**
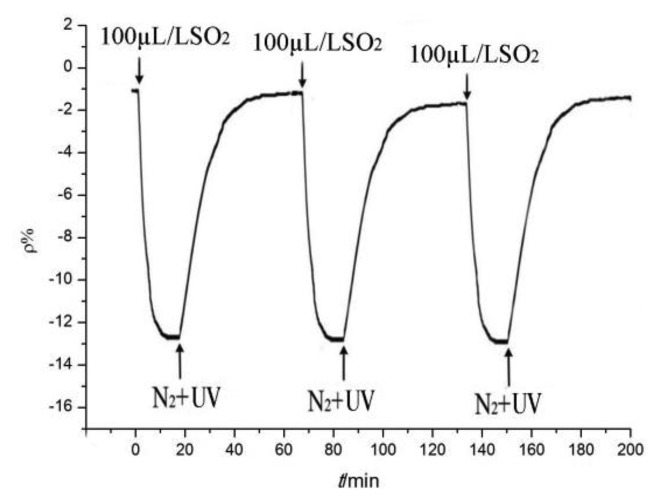
Response and recovery curves.

The sensor with a ratio of 5:1 was selected and used to detect SO_2_ gas to illustrate the desorption and repeatability processes. The tendency of the sensor resistance-change rate is shown in Figure 12.

As shown in Figure 12, the desorption process enables the sensor resistance value to return to the initial value after testing. Many times during the tests, the resistance-change trends remained the same, whereas the maximum change rate remained similar and stable. In sum, the gas sensor may be used repeatedly to detect gases with good stability and reproducibility.

### 3.6. Discussion on the Mechanism of the MWNT Sensor Gas-Sensitive Response

The MWNT film is formed by a number of disordered CNTs, and its resistance model is generally consistent with the heterogeneous filamentous model proposed by Kaiser *et al.* [15]. Thus, two parts determine the total resistance: the resistance value of carbon tube itself, *R*_1_, and resistance value between one tube and another, *R*_2_[15]. That is:

(2) $$R=g_{1}R_{1}+g_{2}R_{2}$$

where $g_{i}=\overset{L_{i}}{\;}/\underset{A_{i}}{\;}$, *i* = 1, 2. *L*_i_ is the conductive effective length of carbon material, and *A*_i_ is the corresponding effective area. In turn, the physical formula of the resistivity can be written as:

(3) $$\rho \left(T\right)=\rho_{1}\left(T\right)+\rho_{2}\left(T\right)$$

where *ρ*_1_ (*T*) is the resistivity of the tube, and *ρ*_2_ (*T*) is the resistivity of the junction.

MWNTs are evenly coated on the electrode surface to form a gas-sensitive film. MWNTs have a tubular structure. The diameter of the tubes ranges from 20 to 30 nm, whereas their length ranges from 1 to 20 μm. Both values are significantly smaller than the electrode spacing of 1 mm. Therefore, the resistance of MWNTs can be considered to reflect the overall conductivity of a plurality of MWNTs that are placed between electrodes. As previously mentioned, adsorbents do not have conductive properties, so the resistance characteristics of sensors are unrelated to the conductivity of the adsorbent. That is, the resistance value of sensors represents the overall conductivity of MWNTs.

The mechanisms by which molecular sieve mixing improves gas response to SO_2_ and H_2_S are as follows: (1) the molecular sieve is porous in structure, similar to MWNTs. The specific surface area is 500 to 1,000 g/m^2^, larger than that of MWNTs which have a specific surface area of approximately 200 g/m^2^. A looser porous structure is conducive for adsorbing more gas molecules on the sensor film, particularly when molecular sieves are mixed to MWNTs. Consequently, more gas molecules are involved in the electron exchange interaction with the sensor gas film; (2) The molecular structures of SO_2_ and H_2_S are polar, and molecular sieves have strong adsorption capacity for polar substances. More gas molecules are present on the sensor film; (3) Some charged metal ions, such as Na^+^, exist in the molecular sieve and may affect MWNT surface charges. These ions could affect the pore size and morphology of MWNTs. More defects could appear on the MWNT surface, which may then enhance the gas adsorption and charge the transfer of sensors; (4) The molecular sieves contain silicon-oxygen bonds, to which the hydroxyl groups can easily attach, so the presence of hydroxyl groups could increase the adsorption of the gas components to a certain extent.

In summary, compared with intrinsic MWNTs, the adsorption capacity and charge transfer capability of adsorbent-mixed MWNT sensors to SO_2_ and H_2_S were significantly improved. The sensitivity and response speeds were also enhanced. In particular, the adsorption capacity of the prepared sensor to SO_2_ increased several times compared with that of the intrinsic MWNT sensor. The sensitivity enhancements of the adsorbent-mixed MWNT sensor to SO_2_ and H_2_S also facilitated the low-concentration detection of these two gases, which is important for the early detection of defects in SF_6_ gas insulated equipment.

## 4. Conclusions

(1) The 4 Å molecular sieve-mixed MWNTs sensors have strong adsorption capacity and high sensitivity to SO_2_ and H_2_S. The tests showed that the adsorbent-mixed MWNT sensors have the highest sensitivity to SO_2_ with a mass ratio 10:1, whereas the adsorbent-mixed MWNT sensors possessed roughly the same high sensitivity to H_2_S with mass ratios of 10:1 and 20:1.
(2) Sensors with different mixing ratios exhibited varied sensitivity responses to SO_2_ and H_2_S. Sensors with different mixing ratios can be selected to achieve the optimum selectivity of the two gases according to this feature. Meanwhile, the gas concentrations and the sensor resistance-change rates followed a linear relationship.
(3) The sensors with adsorbent-mixed MWNTs exhibited good stability to SO_2_ and H_2_S and can be used repeatedly to detect these gases. When UV light was used to desorb residual gas between measurements, the high sensitivity of “poisoned” sensors was restored.
